# Reporting race and ethnicity in aging in place research: A systematic review

**DOI:** 10.1093/geront/gnag146

**Published:** 2026-06-30

**Authors:** Bonnie Albright, Leslie E Green, Denise R McAllister

**Affiliations:** Department of Gerontology, University of Massachusetts Boston, Boston, Massachusetts, United States; School of Family and Consumer Sciences, Texas State University, San Marcos, Texas, United States; School of Family and Consumer Sciences, Texas State University, San Marcos, Texas, United States

**Keywords:** Emergent variable, Structural racism, Discrimination, Logistic regression, Housing

## Abstract

**Background and Objectives:**

Despite differences in housing experience across people from different racial and ethnic groups in the United States, race and ethnicity are not frequently emphasized in aging in place literature. This review cataloged the approach to race and ethnicity (active or passive) taken by quantitative aging in place research articles. This review also tested whether structural research elements were associated with the approach taken.

**Research Design and Methods:**

This study included original research articles with samples of noninstitutionalized older U.S. residents. Included articles addressed an aspect of aging in place related to the physical home, used statistical modeling, and were published between 2014 and 2023. Because an initial database search in this interdisciplinary field did not produce a comprehensive list of articles, the review sample was constructed through manual review of journal tables of contents.

**Results:**

Approximately one third (35.16%) of the 91 sample articles took an active approach to race and ethnicity. The regression model did not detect statistically significant associations between structural research elements and active approaches; however, wide confidence intervals indicate that meaningful effects cannot be ruled out.

**Discussion and Implications:**

Even in the presence of a strong and historic association between race and ethnicity and housing, a passive approach to race and ethnicity was most common. This presents a gap in the existing body of aging in place literature. Practical suggestions for addressing this gap through future research are presented. A multidisciplinary symposium to develop more formal suggestions is recommended.

Most older people desire to age in place, meaning they wish to remain living comfortably and independently in their homes as they age (Centers for Disease Control and Prevention, 2024). However, this can be difficult to achieve, and it can be even more difficult for older people from historically marginalized racial and ethnic groups (Binette & Farago, 2021; Joint Center for Housing Studies of Harvard University, 2023; Ratnayake et al., 2022). Researchers and theorists have identified and studied many aspects of aging in place, including social networks and supports, neighborhood satisfaction, living arrangements, community services, and the accessibility of the physical home (Ahn et al., 2020; Bigonnesse & Chaudhury, 2020; Gitlin, 2003). The physical home is one aspect of aging in place where researchers can draw a direct link to historic discrimination experienced by older people from historically marginalized racial and ethnic groups.

Structural racism is a framework that conceptualizes public and private institutions and actors as operating in concert to codify racial discrimination across multiple societal sectors (Bailey et al., 2017). Within this framework, racial discrimination functions as a fundamental cause of poor health outcomes (Bailey et al., 2021; Link & Phelan, 1995). Structural racism is particularly relevant to aging in place research because housing is among the sectors in which public and private mechanisms have perpetuated racial discrimination. Private decisions of landlords, real estate agents, and neighbors, along with institutional practices of banks and federal policies, have made housing an inequitable environment for many older people (Bailey et al., 2017, 2021).

When an older person identifies with one or more historically marginalized racial and ethnic groups, consideration of intersectionality helps housing scholars and other stakeholders understand how multiple (or overlapping) social identities shape lived experiences and inequities (Bowleg, 2012; Hardeman et al., 2022). According to Bowleg (2012), historically marginalized identities, such as race, ethnicity, gender, and sexual orientation, interact with one another within broader systems of social inequity, including structural racism. These interlocking social identities are not additive but rather form myriad combinations, each with its own context-dependent societal privilege or marginalization. Therefore, recognizing the complexity of an older person’s racial and ethnic backgrounds is critical for identifying barriers to aging in place and developing appropriate interventions (Hardeman et al., 2022; Wallace & Meeks, 2022).

Multiple policies have long made it difficult if not impossible for members of historically marginalized racial and ethnic groups to enjoy housing security, and these difficulties persist today (National Fair Housing Alliance, 2025). Historic discriminatory lending and real estate practices kept potential homeowners away from homeownership, and predatory loan practices in the subprime mortgage crisis disproportionately impacted Black and Hispanic homeowners (Barocas & Selbst, 2016; Castro Baker et al., 2019; Chen et al., 2026; Green & Albright, 2026; Greer, 2013; Keene et al., 2019; Massey et al., 2016). The generational impact of these practices has deprived many Americans of the opportunity to build wealth and pass this wealth to the next generation (Nier, 2007; Rothstein, 2017). These societal barriers to equity-building have contributed to generations of poverty and disadvantage for historically marginalized racial and ethnic groups.

These accumulated disadvantages affect not only access to housing but also the suitability of that housing in later life. A home that does not meet the accessibility needs of an older inhabitant has been termed incongruent housing (Golant, 2012). Incongruent housing can occur as an older person experiences new physical disabilities that often accompany later life (Verbrugge & Jette, 1994). In this way, the home has remained the same, but the inhabitant has changed, and the physical home environment may no longer fit the person (Lawton & Nahemow, 1973). When this happens, the inhabitant may decide to stay and cope with the setting, adapt the setting by modifying it (e.g., adding grab bars, ramps, better lighting), or move (Golant, 2015; Plys, 2019; Pynoos et al., 2012). However, older people from historically marginalized racial and ethnic groups may not have these options due to higher rates of renting, higher housing cost burden, and fewer financial resources (U.S. Department of the Treasury, 2022). Renters must seek permission from landlords to make home modifications, and if permitted, paying for those modifications may be out of financial reach. Many of these older Americans also have higher poverty rates than older White Americans (Cubanski et al., 2018; Shrider & Creamer, 2023), which can mean fewer financial resources for improving the appropriateness of the home environment by either relocating or making adaptive home modifications (Abramson, 2015; Bakk et al., 2017; Epps et al., 2018; Jenkins Morales & Robert, 2020a, 2020b; Robinson-Lane et al., 2023). Because of these financial and housing issues, racially and ethnically marginalized people have fewer options for aging in place.

The difference in disability between older Americans from historically marginalized racial and ethnic groups as compared to older White Americans also differentiates housing trajectories across these groups. Cumulative stressors due to racial discrimination heighten risk through earlier and more frequent onset of disability. Black and Hispanic Americans face more disease burden and have more mobility disabilities in later life as compared to White Americans (Al Snih et al., 2004; Ferraro et al., 2017; Kail et al., 2020; Thorpe et al., 2011; Usher et al., 2021). Functional limitations, including physical mobility limitations, are higher for older Black Americans than they are for older White Americans even with similar numbers of chronic conditions and while controlling for education, income, wealth, and other variables (Kail et al., 2020; Thorpe et al., 2011). These disparities reflect the biological cost of chronic exposure to racism (Boen, 2020; Ferraro & Shippee, 2009; Geronimus et al., 2006). Despite the differences in disease burden, mobility limitations, and financial resources, race and ethnicity are not frequently emphasized in aging in place research (Rosenwohl-Mack et al., 2020).

Literature reviews on race and ethnicity in aging in place are often limited because the experiences of some historically marginalized racial and ethnic groups, including Asian American, American Indian, Alaska Native, Native Hawaiian, Pacific Islander, and multiracial older people, are less frequently documented (Bowleg, 2012; Ng et al., 2014; Robinson-Lane et al., 2023). Studies that have disaggregated commonly merged categories of people found health disparities and resource deprivation among Asian Americans and American Indians (Ng et al., 2014; Whetung & Gonzales, 2025). However, adequate data on older Asian American, American Indian, Alaska Native, Native Hawaiian, Pacific Islander, and multiracial populations are difficult to obtain. These categories are often combined in data collection sources or collapsed into fewer categories (often one) in statistical modeling. Aggregation of data from heterogeneous groups of people masks the socioeconomic and aging in place experiences of people, thus keeping these groups invisible in much of the quantitative research (Boen et al., 2026; Edlagan & Vaghul, 2016; Xiong et al., 2026). This lack of visibility in existing research is itself a form of discrimination, shaping which older people’s experiences become visible in the aging in place literature.

While limited research focuses directly on housing and race and ethnicity in later life (Rosenwohl-Mack et al., 2020), one example of equity-focused aging in place research and practice is the home modification program Community Aging in Place, Advancing Better Living for Elders (CAPABLE) (Spoelstra et al., 2019). CAPABLE provides home modification handyman services plus occupational therapy for older dual-eligible Medicaid and Medicare recipients. As the program was developed, more than 80% of study respondents identified as Black or African American (Szanton et al., 2016, 2018). This program directly benefits those who most need support to age in place. Research that focuses on aging in place for older people from historically marginalized racial and ethnic groups, like the CAPABLE program, can make a meaningful contribution to disrupting the cycle of structural racism in America.

As previously discussed, historic patterns of housing discrimination persist today, and the problem of incongruent housing in later life is worsened by racial and ethnic disparities (Binette & Farago, 2021; Joint Center for Housing Studies of Harvard University, 2023; Ratnayake et al., 2022; Robinson-Lane et al., 2023). However, aging in place research has often engaged only minimally with these differences, treating race and ethnicity as routine demographic variables rather than as substantively meaningful constructs (Rosenwohl-Mack et al., 2020). Efforts to correct inequities in housing infrastructure, financing, and policy offer an opportunity to change this story (Wassmer & Williams, 2021). Because research can influence policy change, housing and aging in place research can play a role in building awareness of this history and supporting equitable improvements. Cataloging how race and ethnicity have been reported in aging in place quantitative research is a step toward that goal.

## Current study

The current study is a systematic review examining the use and treatment of race and ethnicity in aging in place quantitative research published between 2014 and 2023. The first research question for the current study asked what type of approach quantitative aging in place articles took regarding race and ethnicity. The second research question asked if structural research elements were associated with an article’s approach to race and ethnicity. This review also compiled practical applications for the use of race and ethnicity in future articles. While a prior review of qualitative aging in place literature exists (Rosenwohl-Mack et al., 2020), to the authors’ knowledge, this is the first review of how quantitative aging in place articles approach race and ethnicity.

## Method

### Eligibility criteria

The current systematic review study followed the Preferred Reporting Items for Systematic Reviews and Meta-Analyses (PRISMA) reporting guidelines (Page et al., 2021). The PRISMA protocol was registered in PROSPERO (CRD420251022628; see Table S1). Inclusion and exclusion criteria for articles were determined through the Population, Intervention, Comparison, Outcomes, and Study design (PICOS) framework (Methley et al., 2014).

The population (P) of interest was older U.S. residents, their caregivers, or their households. The minimum age threshold was 65, but articles including people ages 55 and older were included when the article primarily addressed issues of aging. This decision reflects a tradeoff: while a lower age bound may introduce heterogeneity in age ranges, these articles contribute information about older people aging in their homes, and to leave them out would reduce the comprehensiveness of the current study. Given that the current study addresses race and ethnicity, the decision to lower the age bound was informed by the allostatic load findings of Geronimus et al. (2006), which indicate that Black Americans may experience age-related physiological decline at earlier chronological ages than White Americans. A lower chronological age threshold therefore captures individuals who may already be experiencing aging-related housing needs.

Because the focus of the current study was on aging in place, articles with samples of people living in institutional settings (e.g., hospitals, nursing care facilities) were excluded. Articles reporting on populations outside the United States were excluded since the current study’s research questions derived from this country’s historic housing trends.

Due to the interdisciplinary nature of gerontology research, intervention types (I) were not restricted. To retain focus on the accessibility of the physical home as an aspect of aging in place that is linked to historic discrimination, articles were included if they referenced the physical home or home safety. Falls studies that included elements of home safety were included because of the link between falls and difficulty aging in place (Stoeckel & Porell, 2010). Falls studies that focused exclusively on other aspects of fall prevention, such as exercise and balance, were excluded.

While most articles did not include a comparison group, articles that studied aging in place with a comparison group (C) were included when the comparison was between those who implemented a factor or intervention and those who did not (e.g., homeowners who completed a home modification for the purpose of aging in place vs those who did not). There were no comparison group exclusions.

Outcome expectations (O) were not limited by inclusion criteria. However, articles that did not include a connection to aging in place were excluded. While often reserved for qualitative reviews, the PICOS component of study design (S) was adapted to focus the search on quantitative original research articles with statistical modeling at the bivariate level or multivariate level. Qualitative articles and review articles were excluded.

### Search strategy

**Figure 1:**
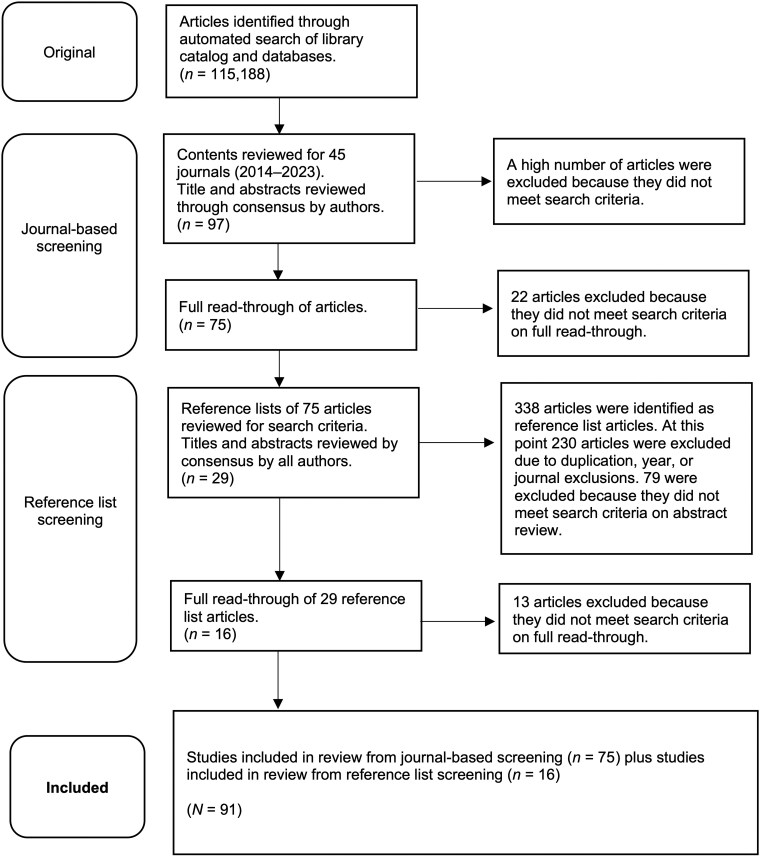
Sample flow diagram.

The researchers originally conducted a library database search, with the assistance of a university librarian, using PICOS criteria terms (see Table S2). The initial PICOS search generated 115,188 articles, which included a high number of unrelated articles from other fields. The authors attempted to focus the search results by selecting related subjects. However, this methodology was abandoned in favor of an inclusive and thorough proactive methodology based on manual review of journals with application of the PICOS framework (Methley et al., 2014) (see Table S3). The final sample for the current systematic review study included 91 articles (see Figure 1).

### Measures

#### Independent variables

Five categories of variables were collected on each article: variables related to bibliographical characteristics of the article, variables related to the article’s data-related elements, variables related to the article’s analytic strategy, variables related to the article’s use of race and ethnicity, and a variable related to the discussion of race and ethnicity in the article.

Bibliographical characteristics of the article included the journal name, the year published, and a binary variable, publish year 2021 or earlier. Articles published in 2021 or earlier were written before *JAMA* and *The Gerontological Society of America* issued new editorial guidance on reporting race and ethnicity (Flanagin et al., 2021; Meeks et al., 2021).

Data-related elements of the articles included data source: the main data source used by the article (name of data source or intervention program) and data type: the type of data used in the article (primary or secondary data source). The article’s sample size was reported, and a binary variable, *smaller sample*, indicated if the article had a sample size less than the median (1 = sample size < 1,052; 0 = sample size ≥ 1,052).

Two measures related to an article’s analytic strategy were collected in the current study. The first variable, *statistical analysis*, captured the most advanced statistical analysis that the article used (e.g., regression, structural equation modeling). The second variable collapsed this list into the variable: *simple statistics*, which was coded as 1 if the article used only a simple statistical method (e.g., chi-square, *t* test). Simple statistics was coded as 0 if the article used an advanced statistical method (e.g., linear or logistic regression, structural equation modeling).

Several measures were created that described how the article addressed race and ethnicity. First, in the *number of categories* variable, the number of racial and ethnic categories used in the article was counted. If an article used collapsed categories (e.g., Asian or Other), each collapsed category was counted as one category. When articles used an *other* category, it was counted as a category in this summation. Second, a *type of race and ethnicity categories* variable summarized this count variable into the following three categories: 0 = article did not use any race or ethnicity categories at all; 1 = article used one or two categories; 2 = article included more than two categories of race and ethnicity. The third race and ethnicity variable, *used in statistics*, asked if the article used race and ethnicity in the statistical modeling or not (0 = no, 1 = yes). Fourth, the *significant model* variable indicated whether race and ethnicity were statistically significant in the article’s model (0 = no, 1 = yes, NA = did not report yes or no). And finally, a fifth variable, *reference group*, listed the reference group that the article used when the statistical analysis required a reference group (e.g., regression).

The discussion variable, *type of discussion*, captured how articles handled discussions of race and ethnicity using this question: “Regardless of whether or not race and ethnicity were used in the model, how much, if at all, were race and ethnicity described in the discussion or limitations section (not findings and not results sections)?” Responses were assigned one of these four mutually exclusive response categories: 0 = not discussed at all in discussion or limitations; 1 = briefly discussed in discussion or limitations: e.g., an add-on to another sentence; 2 = moderately discussed in discussion or limitations: e.g., race and/or ethnicity have their own full simple sentence; 3 = substantively discussed in discussion or limitations: e.g., race and/or ethnicity have at least one complex and thoughtful sentence. If questions arose while coding this variable, it was discussed in detail in weekly meetings until researchers reached coding consensus.

An additional variable from the systematic review process was tested in sensitivity analyses to see if it introduced measurable bias in the methodology. The variable reference-list article reported whether the article was part of the current study’s base sample methodology (see Table S3 Steps 1 through 4) or if it came from the reference-list sample (0 = base sample, 1 = reference-list article; see Table S3 Step 5). This variable was included to test for internal research study bias associated with the dependent variable.

#### Dependent variable

An emergent dependent variable was created for the current study. The emergent variable *approach* assessed the overall approach to race and ethnicity taken by the article. As outlined in the introduction, there is a need for research to robustly recognize and address race and ethnicity issues in aging in place. Two ways articles may practically recognize and address issues of race and ethnicity in aging in place research are through: (a) separate coding of race and ethnicity variables, and (b) narrative discussion in the closing (postresults) sections of the article. A narrative discussion of race and ethnicity could include a range of approaches from addressing limitations in variable measurement and model development to making suggestions for program implementation and policy recommendations. This emergent variable serves as a conceptual benchmark against which sample articles can be evaluated for how robustly they account for and discuss race and ethnicity. Construction of the emergent variable *approach* drew from the litmus test created by Crilly et al. (2023) while remaining within the limited context of the available data collected in the current study.

The *approach* was coded as either passive (0) or active (1). There were two ways that the *approach* variable could be coded as active. First, an article was coded as having an active approach if it treated race and ethnicity separately by having separate coding for race and ethnicity (e.g., if a respondent of Black or African American race and Hispanic ethnicity could be identified in the racial and ethnic categories without being labeled as nonspecific other or non-White). Second, an article could also be coded as active on the *approach* variable if the article moderately or substantively discussed race or ethnicity after reporting findings (i.e., the article scored 2 or 3 on the *type of discussion* variable). An article was coded as active if it met either or both of these conditions. An article was scored as passive on the *approach* variable if it did not use separate race and ethnicity categories and it also did not discuss race and ethnicity after the results section using at least one full sentence. The main dependent variable was coded in this way because it accommodated articles that may have been restricted in reporting race and ethnicity due to (a) journal-level constraints such as word limits or editorial norms discouraging discussion of statistically nonsignificant coefficients, or (b) the underlying nature of race and ethnicity variable construction in the secondary data set.

The variable name *approach* and its response categories of active and passive were chosen to capture active or passive engagement with the topic in as semantically neutral a way as possible while still providing a benchmark for distinguishing which articles recognized and addressed race and ethnicity issues in aging in place (active) from those that did not (passive). The current study’s dependent variable, *approach*, is summarized in the descriptive analysis (Table 1) and used in the logistic regression model (Table 2).

**Table 1:** Univariate and bivariate descriptive statistics by approach variable.

	Overall sample	Passive approach (0)	Active approach (1)	
Variables	*N *= 91^a^	*n *= 59 (64.84%)^a^	*n *= 32 (35.16%)^a^	*p* value^b^
**Bibliographical variables**				
Year article was published	2019 (2016, 2021)	2019 (2016, 2021)	2020 (2016.5, 2021.25)	.30
Publish year 2021 or earlier^c^	74 (81.32%)	50 (84.75%)	24 (75.00%)	.30
**Research elements variables**				
Data type: primary data source	42 (46.15%)	27 (45.76%)	15 (46.88%)	.90
Sample size	1,052 (257, 6,289.50)	765 (166, 5,471.50)	2,044 (384.25, 6,872.25)	.09
Smaller sample^d^	45 (49.45%)	32 (54.24%)	13 (40.63%)	.20
No advanced statistical analysis	19 (20.88%)	12 (20.34%)	7 (21.88%)	.90
**Race and ethnicity variables**				
Number of race and ethnicity categories	3 (2, 4)	2 (1, 3)	4 (3, 5)	<.001***
Article had separate coding for race and ethnicity	13 (14.29%)	0.00 (0.00%)	13 (40.63%)	<.001***
Race and ethnicity used in statistics	53 (58.24%)	29 (49.15%)	24 (75.00%)	.02*
Race and ethnicity significant in statistics^e^	35 (83.33%)	17 (73.91%)	18 (94.74%)	.10
Articles reporting significance	42 (46.15%)	23 (38.98%)	19 (59.38%)	.10
**Discussion variable**				
Type of discussion	0 (0, 1)	0 (0, 1)	2 (1, 3)	<.001***
0 = no discussion	47 (51.64%)	40 (67.80%)	7 (21.88%)	
1 = limited discussion	22 (26.37%)	19 (32.20%)	3 (9.38%)	
2 = moderate discussion	10 (10.99%)	0 (0.00%)	10 (31.25%)	
3 = substantive discussion	12 (13.19%)	0 (0.00%)	12 (37.50%)	
**Current study methodology variable**				
Reference-list article	16 (17.58%)	11 (18.64%)	5 (15.63%)	.70

a Median (IQR); *n* (%).

b Wilcoxon rank sum test; Pearson’s chi-squared test; Pearson’s chi-squared test with Yates’ continuity correction; Fisher’s exact test.

c Articles published in 2021 or earlier were written before editorial guidance on reporting race and ethnicity was published by *JAMA* and *The Gerontological Society of America* (Flanagin et al., 2021; Meeks et al., 2021).

d Articles with a small sample size had a sample size below the median sample size of 1,052.

e Percents are calculated out of total articles in the category that report any significance levels for race and ethnicity variables in statistical models.

* *p* < .05.

*** *p* < .001.

**Table 2:** Logistic regression results predicting active approach to race and ethnicity (*N* = 91).

Model variables	Odds ratio	95% confidence interval	*p* value
Published 2021 or earlier^a^	0.53	(0.18, 1.63)	.26
Data type: primary data source^b^	0.95	(0.38, 2.38)	.92
Simple statistics^c^	1.00	(0.31, 3.02)	>.99
Intercept	0.92	(0.29, 2.89)	.88

a Articles published in 2021 or earlier were written before editorial guidance on reporting race and ethnicity was published by *JAMA* and *The Gerontological Society of America* (Flanagin et al., 2021; Meeks et al., 2021). Variance inflation factor (VIF) = 1.05.

b Primary data source = 1, secondary data source = 0. VIF = 1.11.

c Articles were coded simple (1) if they used only *t* tests, chi-square, or ANOVA analysis. Article models were coded advanced (0) if they used regression, structural equation modeling, or other higher level statistical modeling. VIF = 1.08.

A random sample of 22 articles was double coded by two researchers (see Table S6). Variables in the regression model had kappa scores between 0.79 and 1.00 (*p* < .001), reflecting substantial to almost perfect agreement (Cohen, 1960; Landis & Koch, 1977). Risk-of-bias assessment for individual articles followed PRISMA 2020 reporting recommendations (Page et al., 2021) (see Table S1). PRISMA Item 11 addresses whether design or conduct features of each article may have biased its own findings. Because the current study did not extract or synthesize the substantive findings of sample articles, but rather coded how each article reported and discussed race and ethnicity, a formal assessment of risk-of-bias in article findings was not applicable to the research questions.

### Analytic strategy

Univariate and bivariate descriptive statistics were run to describe the sample of articles both in general and by the *approach* variable. A logistic regression model was estimated with *approach* as the dependent variable. The independent variables in this logistic regression were *year 2021 or earlier* (reference group: published 2022 or later), data type (reference group: secondary data source), and simple statistics (reference group: advanced). The *smaller sample* variable showed correlation with data type and was thus left out of the logistic regression. The *journal name* variable was run in sensitivity analyses and found not to be statistically significant; given the complexity this large categorical variable introduced to the analysis, it was left out of the final analysis. The *reference-list article* variable was also tested in sensitivity analysis and found not to be statistically significant; because this variable provided an internal validity test and was not related to the current study’s research questions, it was left out of the final analysis.

## Results

### Univariate and bivariate descriptive statistics

Of the 91 articles in the current study, the average year of publication was 2019 (*SD* = 2.89 years, range 2014–2023; see Figure S1), with 81.32% of the articles published in 2021 or earlier (see Table 1). The final sample comprised articles from 32 separate journals, with 18.68% published in the *Journal of Applied Gerontology* and 16.49% from *The Gerontological Society of America*’s publications (*The Gerontologist* [7.69%], *The Journals of Gerontology: Series A* [4.40%], and *The Journals of Gerontology: Series B* [4.40%]), 10.99% from the *Journal of the American Geriatrics Society*, and the remainder spread across the other journals (see Table S5).

The data in these articles were obtained through primary data collection (46.15%) and secondary data sources (53.85%). Three secondary data sets together accounted for 29.67% of the overall sample of articles: the National Health and Aging Trends Study (NHATS; 15.38%), the American Housing Survey (AHS; 7.69%), and the Health and Retirement Study (HRS; 6.59%). Because articles may be limited by the underlying coding of the secondary data, it is important to understand the nature of this coding. The NHATS initially codes multiple race and Hispanic ethnicity responses when interviewing respondents but then collapses these responses into a single derived variable (racehisp) that combines race and Hispanic ethnicity (Kasper & Freedman, 2020). Researchers using the NHATS who wish to use race and Hispanic ethnicity as separate variables must use underived data. If these articles used the derived (combined single) variable, they were still categorized as active if they included at least one full sentence about race and ethnicity in the discussion or limitations section. Both the AHS and the HRS have separate variables for race and Hispanic origin (Health and Retirement Study, 1992, 2025; U.S. Census Bureau, 2026). Articles using the AHS and the HRS were not limited by the underlying coding of the data and could achieve active status by either using the secondary data source’s original coding methods or by discussing race or ethnicity in their discussion section.

The median article sample size was 1,052, with a generally bimodal response between those using original data collection and those using large representative samples. Most articles used *t* tests and chi-square statistical testing of univariate and bivariate comparisons. More advanced statistical methods, such as regression and structural equation modeling, were additionally used in 79.12% of the articles.

Fourteen articles (15.38%) did not report any race or ethnicity categories. Of the remaining 84.62% of articles, the number of race and ethnicity categories reported ranged from 1 to 9 and was generally normally distributed with a mean of 3.34 categories (*SD* = 1.49). More than half of articles (*n *= 53, 58.24%) used race or ethnicity in a statistical analysis. Of the 42 articles that reported significance levels, 83.33% (*n *= 35) found race or ethnicity statistically significant. In the 35 articles that reported a statistical reference group for their race or ethnicity variables, 80% (*n *= 28) used a White or non-Hispanic White group.

Thirteen articles (14.29%) reported race and ethnicity separately. On the *type of discussion* variable, which ranged from 0 to 3, the mean score was 0.82 (*SD* = 1.04). Just over half of the articles (51.64%) had no discussion of race and ethnicity. On the *approach* variable, approximately one third (35.16%) of the articles scored as active (i.e., indicating that the article either used separate coding of race and ethnicity variables or provided at least one full sentence in the discussion or limitation section about race or ethnicity or both). The remaining articles scored as passive (64.84%; i.e., indicating that the article lacked any active *approach* criteria).

#### Comparisons of groups

Table 1 also describes the current study article characteristics across the dependent variable. The active *approach* column reports characteristics of articles coded as active *approach*, and the passive column reports characteristics of articles coded as passive *approach*. Four variables indicated statistical differences across these subgroups. First, articles with an active *approach* had a higher median number of race and ethnicity categories (4 vs 2; *p* < .001). Second, articles with an active *approach* were more likely to have separate coding for race and ethnicity variables (*p* < .001). Third, articles with an active *approach* had a higher median type of discussion (2 vs 0; *p* < .001). It would be expected that these variables differ across the subsamples, because these variables were used in the construction of the emergent *approach* variable (see Measures section). Lastly, race and ethnicity were more often used in the statistical models of articles with an active as compared to passive *approach* (75.00% vs 49.15%; *p* = .02). No additional bibliographic, data and analytic, or race and ethnicity variables showed statistically significant differences across these subgroups of active versus passive *approach*.

Two statistically significant bivariate correlations were identified between independent variables. Data type and a smaller sample showed a positive and statistically significant correlation, *r*(89) = 0.58, *p* < .001. To reduce the possibility of multicollinearity between these variables, a smaller sample was not included in the logistic regression analysis. Data type and simple statistics model were also correlated, *r*(89) = 0.28, *p* = .006.

### Logistic regression results

A logistic regression model (Table 2) tested the research question: are structural research elements associated with aging in place articles’ approach to race and ethnicity? The two data and analysis elements tested were data type and simple statistics. The model was also conditioned on if the article had a publish year of 2021 or earlier.

Article publication date of 2021 or earlier was not statistically significant in the analytic model (OR = 0.53, 95% CI [0.18, 1.63], *p* = .26). Neither the primary versus secondary data source variable (OR = 0.95, 95% CI [0.38, 2.38], *p* = .92) nor the simple versus advanced statistical method variable (OR = 1.00, 95% CI [0.31, 3.02], *p* > .99) was statistically significant in the model. With three predictor variables and 32 of 91 articles (35.16%) categorized as having an active *approach*, the model has limited statistical power, and the wide confidence intervals suggest that meaningful effects cannot be ruled out. Multicollinearity testing indicated that variance inflation factor (VIF) values were below conventional thresholds for all predictor variables (O’Brien, 2007). VIF values for individual predictors are reported in Table 2.

## Discussion

The first research question for the current review asked what type of approach quantitative aging in place articles published between 2014 and 2023 took regarding race and ethnicity. A passive approach meant that an article neither treated race and ethnicity separately nor did it discuss race or ethnicity after reporting findings. This research found most of the sample articles took a passive approach. This result from the aging in place literature examined in this systematic review is telling; even in the presence of a strong and historic association between race and ethnicity and housing, a passive approach was most common. Indeed, each research article had its own focus and aim, but very few acknowledged the underlying role of race and ethnicity in incongruent housing for aging in place. This omission presents both a gap and a disadvantage in the existing body of aging in place literature.

A second research question asked if structural research elements, such as data type and simple or advanced statistical model used, were associated with an article’s approach to race and ethnicity. This question acknowledged the possibility that structural research elements of the data type or statistical model could have predetermined what approach to race and ethnicity was analytically possible for the article. A statistically significant finding would have indicated that research elements were associated with approach. The regression model did not detect statistically significant associations between structural research elements and approach to race and ethnicity; however, wide confidence intervals indicate that meaningful effects cannot be ruled out.

These findings are based on ten years of quantitative aging in place research across multiple disciplines. While the authors’ intention to include as many articles as possible in the review may have limited the depth of the findings, the simple breadth of the findings is informative. Two-thirds of articles did not engage in active reporting on race and ethnicity and instead side-stepped this complicated issue. One possible reason for this may be that publishers discourage discussion of statistically nonsignificant findings, so if race and ethnicity variables are not significant in the article’s statistical models, then race and ethnicity might not be discussed beyond reporting results. However, insofar as race and ethnicity are important elements in the history of housing in the United States, it is arguably important to include and discuss race and ethnicity regardless of statistical significance. Due to the historic inequities in housing in the United States, it may be myopic for aging in place researchers to ignore the ways that race and ethnicity are at work in their analyses.

### Limitations

Several limitations to the current study should be noted. The current study sample was drawn from highly rated U.S. peer-reviewed journals in four disciplines. Increasing the scope of this study to international peer-reviewed journals as well as gray literature sources could contribute to a more complete picture. The inclusion of reports from government and other organizations as well as industry and consumer journals could improve contextualization of the story of reporting race and ethnicity in aging in place literature.

Another limitation is that a risk-of-bias assessment of missing articles or results was not conducted. Item 14 of the PRISMA 2020 checklist concerns bias in a review due to missing articles or missing results within an article (Page et al., 2021) (see Table S1). Articles may be missing from this review because of omission due to human error (i.e., the authors did not recognize an article that should have been included) or due to nonpublication. Nonpublication occurs when researchers choose not to submit findings or when journals preferentially select articles (i.e., studies with statistically significant findings). Also, results may be missing within published articles because articles report only statistically significant findings. The current study briefly discusses potential nonreporting of statistically nonsignificant race and ethnicity coefficients in sample articles but does not formally assess this aspect of risk-of-bias. Articles mistakenly not included in the sample, selective nonpublishing of findings, and nonreporting of statistically nonsignificant coefficients in the source literature could affect the summary of reporting practices presented here.

Sixty-nine (75.82%) of the articles were single-coded, which introduced a source of potential weakness in the data. The authors met regularly through the research process to discuss coding concerns. When ambiguous coding situations were brought to the group by one author, the other two authors blind-coded on the spot, and a final decision was made by consensus. However, most of the articles were coded by one author, and this introduced potential bias inherent in single-coded data.

Some articles were limited by the categorizations of race and ethnicity in the underlying secondary data source. These articles could still score as active if they provided a full sentence about race or ethnicity in the discussion or limitations section of the paper, but these reporting practices were downstream of data set design choices. Sample articles that used a secondary data set with a combined race and ethnicity variable rather than separately coded race and ethnicity variables may have been unfairly penalized in the analysis because one of the pathways to achieving an active approach was not available to them.

Finally, there is a limitation in the analysis regarding power. Due to the wide confidence intervals in the logistic regression coefficients, the data cannot rule out meaningful effects. A formal power analysis could determine a minimum sample size for improving interpretability of the regression results.

### Implications

While the goal of this review was to share findings on the current state of the use of race and ethnicity in aging in place research, practical implications can also be drawn. Future research may consider the following strategies:

- Talk about it. Due to the theoretical and explanatory value of race and ethnicity variables in housing research, discuss these variables regardless of whether or not they are found to be statistically significant. If it is not possible to include race and ethnicity variables in the research, then explain why this is the case. For example, if the sample is predominantly White, name this constraint explicitly in the limitations section and discuss what may be missing regarding aging in place experiences for older people from historically marginalized racial and ethnic groups that were not included in the study.
- Separate race and ethnicity variables when possible. This has been a recommendation from guidance in highly regarded journals (Flanagin et al., 2021; Meeks et al., 2021), and it allows for findings across both race and ethnicity. In practice, when working with primary data, this means coding race and ethnicity as distinct variables rather than collapsing them into a single combined race/ethnicity variable. When working with secondary data, this may mean retaining the original separate race and ethnicity variables rather than using derived variables that combine them (e.g., the original race and Hispanic ethnicity variables in NHATS) (Kasper & Freedman, 2020).
- Run separate models or use interactions. If the theory and prior research support separate explanatory models by race and ethnicity, consider running separate models or using interaction effects (Suntai et al., 2024). Statistical approaches such as penalized regression and multilevel modeling with partial pooling are available for analyzing smaller subgroups, and researchers could consult with a statistician to identify the appropriate approach for their study (Bafumi & Gelman, 2007; Firth, 1993; Heinze & Schemper, 2002; Much, 2025). Additionally, sensitivity analyses can be reported in the supplementary materials of an article, if required, to meet publication word count limits.box

This research also supports a call to action for the research community. The above practical implications are a starting point for a larger conversation. More formal suggestions may be taken up by a symposium of scholars on the topics of race and ethnicity and housing, as has been done on other gerontology topics (Kelley & Thorpe, 2023; Walston et al., 2019). A community scholar effort from housing researchers, gerontologists, race and ethnicity scholars, and statisticians could provide a platform for agreement on protocols for navigating this integrated topic and build consensus-driven guidelines to inform aging in place research.

Possible concrete deliverables from such a collaborative interdisciplinary symposium include: (a) a consensus checklist elaborating the basic list of recommendations above; (b) model language for journal submission guidelines that explicitly encourages the discussion of race and ethnicity in the discussion and limitations sections of article submissions regardless of their coefficient significance in statistical models; (c) a decision tree for authors working with constrained data sets that provides a scaffold for researchers navigating the symposium recommendations; and (d) a community research agenda that builds on findings from the current study and similar work to identify gaps and suggest a path forward for more equitable engagement with race and ethnicity in aging in place research.

Research on race, ethnicity, and aging in place can be directly applicable to policy implementation and lead to three key benefits. First, statistical modeling becomes better aligned with theoretical and historical contexts. Second, these considerations contribute to greater equity in aging and housing research (Suntai et al., 2024). Third, research that accounts for the persistent effects of historic inequities in housing infrastructure, financing, and policy offers opportunities for equitable improvement in aging in place (Wassmer & Williams, 2021). The historic association between race and ethnicity and housing in the United States illustrates that these factors are important parts of any aging in place researcher’s work.

## Supplementary Material

gnag146_Supplementary_Data

## Data Availability

This systematic review was pre-registered in PROSPERO (CRD420251022628). This study analyzed previously published research and did not require institutional review board approval. The article coding data and coding scheme that support the findings of this study are not publicly available because the authors have not completed planned analyses for future publications; however, they are available from the corresponding author upon reasonable request.
