# Supplementary material for: Reporting race and ethnicity in aging in place research: A systematic review
Source: Gerontologist. 2026 Jun 30;66(8):gnag146. doi: 10.1093/geront/gnag146 (PMC13389311; doi:10.1093/geront/gnag146)
Supplement: gnag146_Supplementary_Data [file gnag146_supplementary_data.pdf]

Reporting Race and Ethnicity in Aging in Place Research

A Systematic Review

Bonnie Albright[1], Leslie E. Green[2], and Denise R. McAllister[3]

[1]Department of Gerontology, University of Massachusetts Boston

[2]School of Family and Consumer Sciences, Texas State University

[3]School of Family and Consumer Sciences, Texas State University

Author Note

Corresponding Author: Bonnie Albright, Ph.D. https://orcid.org/0000-0003-2827-4444 Department of Gerontology, University of Massachusetts Boston, Boston, MA USA. Email: bonnie.albright001@umb.edu

Leslie E. Green, Ph.D. https://orcid.org/0000-0002-6105-3128 School of Family and Consumer Sciences, Texas State University, San Marcos, TX USA. Email: lgreen@txstate.edu

Denise R. McAllister, Ph.D., School of Family and Consumer Sciences, Texas State University, San Marcos, TX USA. Email: dr.mca@txstate.edu

We have no conflict of interest to declare.

Funding

None reported.

Acknowledgments

This systematic review was pre-registered in PROSPERO (CRD420251022628). This study analyzed previously published research and did not require institutional review board approval. The article coding data and coding scheme that support the findings of this study are not publicly available because the authors have not completed planned analyses for future publications; however, they are available from the corresponding author upon reasonable request.

SUPPLEMENTARY MATERIALS

Supplementary Table S1

PRISMA 2020 Checklist[a]

Section and topic	Item #	Page #
TITLE		
Title	1	1
ABSTRACT		
Abstract	2	3
INTRODUCTION		
Rationale	3	5–9
Objectives	4	9–10
METHODS		
Eligibility Criteria	5	10
Information Sources	6	58
Search Strategy	7	57, 58
Selection Process	8	59
Data Collection Process	9	58–60
Data Items (outcomes)	10a	12
Data Items (other variables)	10b	12
Study Risk of Bias Assessment	11	16
Effect Measures	12	14
Synthesis Methods (eligibility for synthesis)	13a	15
Synthesis Methods (preparing for synthesis)	13b	15
Synthesis Methods (tabulation and graphical methods)	13c	16
Synthesis Methods (statistical synthesis methods)	13d	16
Synthesis Methods (methods to explore heterogeneity)	13e	16
Synthesis Methods (sensitivity analyses)	13f	16
Reporting Bias Assessment	14	22
Certainty Assessment	15	15
RESULTS		
Study Selection (flow of studies)	16a	53
Study Selection (excluded studies)	16b	11
Study Characteristics	17	27
Risk of Bias in Studies	18	16

Results of Individual Studies	19	17
Results of Syntheses (characteristics of contributing studies)	20a	51
Results of Syntheses (results of statistical syntheses)	20b	52
Results of Syntheses (result of investigations of heterogeneity)	20c	18
Results of Syntheses (results of sensitivity analyses)	20d	16
Reporting Biases	21	22
Certainty of Evidence	22	23
DISCUSSION		
Discussion (Interpretation)	23a	20
Discussion (limitations of evidence)	23b	22
Discussion (limitations of review processes)	23c	21
Discussion (implications)	23d	23
OTHER INFORMATION		
Registration and Protocol (registration)	24a	10
Registration and Protocol (protocol)	24b	10
Registration and Protocol (amendments)	24c	10
Support	25	2
Competing Interests	26	2
Availability of Data, Code and Other Materials	27	2

[a] Based on checklist as described in (Page et al., 2021).

Supplementary Table S2

PICOS[a] Search Terms Used in Library Catalog Sample Selection Step

PICOS steps	Included terms/procedure
Population	(“aging” OR “aged” OR “older” OR “older people” OR “older adult” OR “older adults” OR “senior” OR “seniors” OR “generation” OR “silent generation” OR “baby-boomer” OR “baby-boomers” OR “baby boomer” OR “baby boomers” OR “elder” OR “elders” OR “elderly” OR “geriatric”)
Intervention	(“age in place” OR "age-in-place" OR "aging in place" OR "aging-in-place" OR “independent aging” OR “aging independently” OR "transition" OR “transitions” OR “transitioning” OR "move" OR “moves” OR “moving” OR “downsize” OR “downsizing” OR “housing transition” OR “housing adaptation” OR “housing adaptations” OR "residential" OR "housing" OR "residence" OR “home” OR “environment” OR “residential satisfaction” OR “community satisfaction” OR "housing tenure" OR "years in the home" OR “P-E Fit” OR “person-environment fit” OR “environmental press” OR “residential normalcy” OR “ecological theory of aging”)
Comparison	NA
Outcome	Outcome expectations (O) were not limited by inclusion criteria. Articles that did not include a connection to aging in place were excluded.
Study design	(“quantitative” OR “model”)
Search location	University of Massachusetts Healey Library Databases including<br>• APA PsycARTICLES (EBSCOhost)<br>• CINAHL Ultimate<br>• DOAJ Directory of Open Access Journals<br>• EBSCOhost Academic Search Complete<br>• Elsevier ScienceDirect Journals Complete<br>• Gale Academic OneFile<br>• Gale Academic OneFile Select<br>• Gale General OneFile<br>• Gale OneFile: Health and Medicine<br>• Gale OneFile: Nursing and Allied Health<br>• Gale OneFile: Psychology<br>• IngentaConnect Journals<br>• Journals@Ovid Ovid Autoload<br>• MEDLINE<br>• Oxford University Press Journals Current<br>• ProQuest Central<br>• PubMed Central<br>• Single Journals<br>• Web of Science
Search date	September 7, 2023
Results	115,188

[a](Methley et al., 2014)

Supplementary Table S3

Sample Selection Steps

Step	Description
1	***Journals identified.*** The authors chose four disciplines where peer-reviewed aging in place articles would be published: architecture, gerontology, housing, and interior design. From this list of disciplines, the first author generated a list of all peer-reviewed journals using the University of Massachusetts Healey Library Browzine catalog in these disciplines along with their 2022 Clarivate journal impact factor rating.<br>Note: The authors acknowledge that international journals may periodically publish research examining U.S.-based samples. However, because U.S. samples are not the primary focus of these journals, international journals were excluded from the current study. Research applying aging in place concepts to global contexts may warrant a separate review.<br>*The authors chose four disciplines where peer-reviewed aging in place articles were traditionally published: architecture, gerontology, housing, and interior design. Using these disciplines, the first author used the Browzine database through the University of Massachusetts Healey Library to generate a list of 146 journals along with their journal impact factor ratings. The first author reviewed the aims and scope section of each journal, and removed journals that were not peer reviewed, were no longer in print, were student journals, had an international focus, and that were within the target disciplines but grossly out of scope for the current research. The list of journal names was reviewed by each author and based on their professional knowledge three additional journal titles were added (see Supplementary Table S4).*

2	***Journal contents reviewed.*** The journals were distributed evenly across the authors, and the tables of contents for each journal in the ten years of 2014 through 2023 were reviewed for relevance to this study and collected in Zotero.<br>*In the second step, the journals were distributed evenly across the authors, and the tables of contents for each journal issue over a 10-year period (2014–2023 inclusive) were reviewed for research articles relevant to this study. The review included a title review followed by an abstract review if necessary; full text .pdfs of articles appearing to meet the PICOS criteria were saved for further review in Step 3. If there was any question about whether an article met the inclusion criteria the authors erred on the side of caution and included the article.*
3	***Abstract reviewed.*** The authors conducted weekly meetings and together conducted abstract reviews to determine if the original PICOS terms were sufficiently met to justify a full read-through.<br>*In the third step, the authors completed a review of the abstract of questionable articles to determine if the PICOS terms were sufficiently met to justify a full read-through of the article. The authors met weekly during this time to discuss questionable abstracts for inclusion; decisions were made by consensus.*
4	***Full read-through and data collection.*** Full read-through of articles was conducted. At this time, the authors determined if the article was to remain in the study and collected data using a uniform data collection spreadsheet.<br>*In the fourth step, the articles were divided among the first 2 authors who fully read each article and collected data on each article. Several articles were removed that, on close inspection during the full read-through, did not meet all PICOS terms.*

	Meetings between the authors occurred when the inclusion of an article was questionable and the decision to include or drop was made based on consensus.
5	***Reference lists reviewed.*** The authors went through the reference lists of each article in the base sample to identify additional relevant articles. Articles identified from reference lists were subjected to the same review process starting again with Step 3.<br>*In the fifth step, the authors went through the reference lists of the articles in the base sample (that was created through Steps 1–4) to identify additional relevant articles published during the period of review (2014–2023) for inclusion in the final sample. Articles were removed if they were duplicates, appeared in journals that had already been reviewed in Step 2, were from international journals, were from journals no longer published, were from non-journal sources or from non-peer reviewed journals, or were not original research. The remaining articles were then subjected to the same review process described in Step 3 above which resulted in the selection of articles for a full read-through. These articles were distributed among the first two authors for a full read following the process outlined in Step 4 above. The final sample therefore included articles from the original search plus articles generated from the reference list review.*

Supplementary Table S4

Journals and Volumes Reviewed

Journal names	Volumes reviewed
Adultspan Journal	1–22
Aging	5–15
Aging, Neuropsychology, and Cognition	20–30
Anthropology & Aging	35–44
Architecture: Media Politics Society	2–26
BioMed Central (BMC) Geriatrics	13–23
Clinical Gerontologist	36–46
Design Issues	29–39
Enquiry: The ARCC Journal of Architectural Research	10–20
Frontiers in Aging (first published in 2020)	1–4
Geriatric Nursing	34–54
Geriatric Orthopaedic Surgery & Rehabilitation	4–14
The Gerontologist	53–63
Health Environments Research & Design Journal (HERD)	6:2–16
The Historic Environment: Policy & Practice	4–14
Home Cultures	10–20
Housing and Society	40–50
Housing Policy Debate	23–33
Housing Studies	28–38
Interiors: Design, Architecture and Culture (Volume 13 was published in 2024 and thus not reviewed)	4–12
Journal of Aging and Environment (formerly Journal of Housing for the Elderly)	27–37
Journal of Aging and Health	25–35
Journal of Aging and Physical Activity	21–31
Journal of Aging & Social Policy	25–35
Journal of Aging Research	2013–2023
Journal of Aging Studies	27–67
Journal of Applied Gerontology	32–42
Journal of Architecture and Urbanism	37–47
Journal of Gerontological Social Work	56–66
Journal of Housing Economics	22–62
Journal of Interior Design	38–48
Journal of Medical Internet Research (JMIR) Aging (Journal started publishing in 2018)	1–6
Journal of the American Geriatrics Society	61–71
Journal of the American Medical Directors Association	14–24
The Journals of Gerontology: Series A	68–78
The Journals of Gerontology: Series B	68–78

Physical & Occupational Therapy in Geriatrics	31–41
Planning Perspectives	28–38
Research on Aging	35–45
Space and Culture	16–26
The Structural Design of Tall and Special Buildings	22–32
Topics in Geriatric Rehabilitation	29–39

Note. Review window: 2014–2023 (10 years). Volume 2013 was screened during pilot review but excluded from the final analysis.

Supplementary Table S5

Journals in Final Sample

Journal names	*n* (%)
American Journal of Public Health	1 (1.10%)
Archives of Gerontology and Geriatrics	2 (2.20%)
Archives of Physical Medicine Rehabilitation	1 (1.10%)
BMC Geriatrics	2 (2.20%)
Community Development	1 (1.10%)
Family and Consumer Sciences Research Journal	1 (1.10%)
The Gerontologist	7 (7.69%)
HERD: Health Environments Research & Design Journal	3 (3.30%)
Housing and Society	5 (5.49%)
Housing Policy Debate	3 (3.30%)
Housing Studies	1 (1.10%)
Injury Epidemiology	1 (1.10%)
Injury Prevention	1 (1.10%)
JAMA Internal Medicine	1 (1.10%)
JAMA Network Open	1 (1.10%)
Journal of Aging and Environment (formerly: Journal of Housing for the Elderly)	1 (1.10%)
Journal of Aging and Health	3 (3.30%)
Journal of Applied Gerontology	17 (18.68%)
Journal of Gerontological Social Work	4 (4.40%)
Journal of Health Disparities Research and Practice	1 (1.10%)
Journal of Housing Economics	1 (1.10%)
Journal of Housing for the Elderly (later: Journal of Aging and Environment)	5 (5.49%)
Journal of Interior Design	1 (1.10%)
Journal of the American Geriatrics Society	10 (10.99%)
Journal of the American Planning Association	1 (1.10%)
Journal of Urban Economics	1 (1.10%)
Journal of Urban Health	1 (1.10%)
The Journals of Gerontology: Series A	4 (4.40%)
The Journals of Gerontology: Series B	4 (4.40%)
New England Journal of Medicine	1 (1.10%)
Ophthalmology	1 (1.10%)
Physical and Occupational Therapy in Geriatrics	2 (2.20%)
Research on Aging	2 (2.20%)
	***N* = 91**

Supplementary Table S6

Interrater Reliability

Variables	*n* pairs	Coding agreement	κ	
Year published[a]	22	100.00%	1.00	***
Data type[a] (primary, secondary, both)	22	90.91%	.79	***
Sample size[b]	22	63.64%	.62	***
Simple or advanced statistics[a]	22	100.00%	1.00	***
Number of categories	22	90.91%	.88	***
Article had separate coding for race and ethnicity	22	100.00%	1.00	***
Race and ethnicity used in models[c]	22	86.36%	.68	***
Race and ethnicity significant in statistical models[c]	22	68.18%	.47	**
Type of discussion	22	100.00%	1.00	***
Approach[d]	22	100.00%	1.00	***

*$p < .05$, ** $p < .01$, *** $p < .001$

[a] Variable used in logistic regression model.

[b] There were some discrepancies in sample size as some studies reported both weighted and unweighted sample sizes.

[c] There were some discrepancies because information appeared only in supplementary materials.

[d] The *approach* variable was derived from coding for race and ethnicity and type of discussion variables.

Supplementary Figure S1

Histogram of Articles by Year

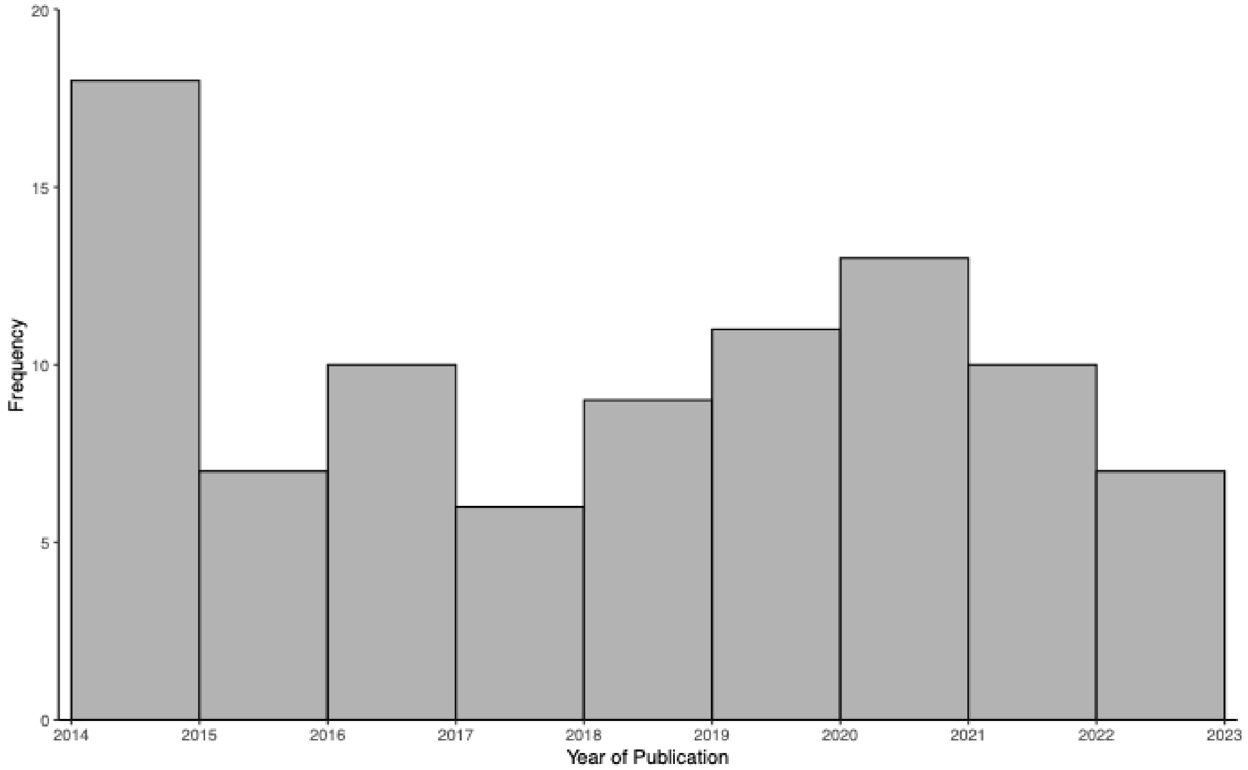

Alt text: Histogram showing the distribution of publication years across the 91 articles included in the systematic review. Publications span 2014 to 2023, with frequency on the y-axis ranging from 0 to 20. The 2014 cohort is the largest at 18 articles. Publication counts dip to 6 articles in 2017 before rising through the late 2010s and early 2020s, reaching a secondary peak of 13 articles in 2021. Counts decline modestly in 2022 (10 articles) and 2023 (7 articles).
